# The bacterial Type III toxin-antitoxin system, ToxIN, is a dynamic protein-RNA complex with stability-dependent antiviral abortive infection activity

**DOI:** 10.1038/s41598-017-18696-x

**Published:** 2018-01-17

**Authors:** Francesca L. Short, Chidiebere Akusobi, William R. Broadhurst, George P. C. Salmond

**Affiliations:** 1 Department of Biochemistry, Hopkins Building, Downing Site, Tennis Court Road, University of Cambridge, Cambridge, CB2 1QW UK; 2 Present Address: Wellcome Trust Sanger Institute, Genome Campus, Hinxton, CB10 1SA UK; 3 Present Address: Department of Immunology and Infectious Diseases, Harvard T. H. Chan School of Public Health, Boston, Massachusetts USA

## Abstract

Bacteria have evolved numerous defense systems to protect themselves from viral (bacteriophage) infection. The ToxIN system of *Pectobacterium atrosepticum* is a Type III toxin-antitoxin complex and “altruistic suicide” anti-phage system, which kills phage-infected cells through the release of a ribonuclease toxin, ToxN. ToxN is counteracted by a co-transcribed antitoxic RNA pseudoknot, ToxI, which self-assembles with ToxN into an inactive 3 ToxI:3 ToxN complex *in vitro*. However it is not known whether this complex is predominant *in vivo*, or how the complex is disassembled following infection to trigger a lethal, “altruistic” response. In this study, we characterise ToxI turnover and folding, and explore the link between complex stability and anti-phage activity, with a view to understanding events that lead to ToxN-mediated suicide following phage infection. We present evidence that ToxN constantly cleaves fresh ToxI *in vivo* rather than staying associated with pre-processed antitoxin, and that the ToxI antitoxin can partially fold spontaneously using conserved nucleotides. We also show that reducing the stability of the ToxIN complex can increase the strength of the antiviral response in a phage-dependent manner. Based on this information, we propose a revised model for ToxN inhibition, complex assembly and activation by infecting bacteriophage.

## Introduction

Bacteria are constantly threatened with predation by bacteriophages, which are estimated to outnumber their bacterial hosts by ten to one^1^. In response, bacteria have evolved several defense mechanisms to protect themselves against phage infection. These mechanisms can be subsequently countered by evolved phage mutants, resulting in an “arms race” of antagonistic co-evolution of bacteria and phage^2,3^. Bacteriophage resistance mechanisms include adsorption inhibition, superinfection exclusion, cleavage of phage nucleic acids through restriction-modification or CRISPR-Cas systems, and abortive infection (Abi)^3,4^. While the majority of these mechanisms protect at the single-cell level, abortive infection systems protect populations of bacteria by triggering the premature death of phage-infected cells, before the phage can replicate and spread. Abortive infection can therefore be seen as a last-resort “altruistic suicide” on the part of an infected cell that protects clonal siblings.

Abortive infection requires a toxic or bacteriostatic product together with a mechanism to protect the host cell from its activity under normal conditions. Unsurprisingly, there is a link between Abi and toxin-antitoxin (TA) systems. TA systems are extremely prevalent genetic modules in bacteria with multiple reported roles including plasmid stabilisation, resistance to phage, and growth arrest in response to various environmental cues^5,6^. A canonical TA system comprises the genes for the antitoxin and toxin arranged in a bicistronic operon. Because antitoxins are relatively unstable, continued synthesis of both components is required to hold the toxin in check; a sudden reduction in expression (due to physiological conditions, or loss of the TA locus) will therefore passively activate the toxin. While toxins are always proteins, antitoxins can be proteins or RNAs^5^. Type I antitoxins are RNAs which prevent toxin translation; Type II, IV, V and VI systems utilise protein antitoxins, with different modes of action^5^. The Type III systems have structured RNA antitoxins, which inhibit their toxin protein partners by direct binding^7–9^. Several TA systems have anti-phage activity including Hok-Sok (Type I)^10^, LsoAB and MazEF (Type II)^11,12^, AbiEG (Type IV)^13^, and ToxIN (Type III)^7^. Type II TA systems are horizontally transferred and are frequently found together with other phage defense genes^14^.

**Figure 1:**
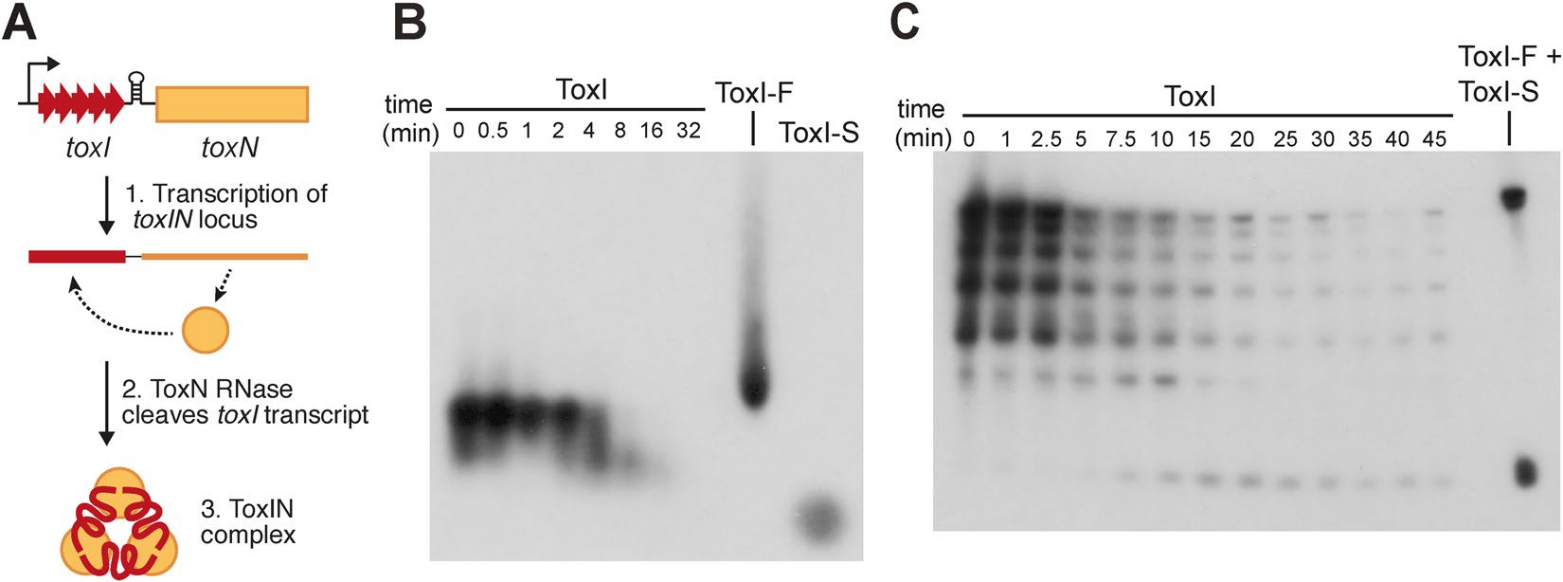
ToxIN half-life *in vivo*. (**A**) Schematic of the ToxIN locus. The *toxI* and *toxN* genes are co-transcribed, with ToxI produced in excess due to the transcriptional terminator preceding *toxN*. The ToxN protein cleaves the repetitive ToxI transcript and assembles into a triangular complex of 3 processed ToxI repeats and 3 ToxN monomers. (**B**) Northern blot for ToxI in *E. coli* DH5α using a DIG-labelled probe. Time points following rifampicin addition are indicated. At t = 0 the majority of the ToxI is full-length, and this is degraded over the course of the experiment. Controls of *in vitro*-transcribed ToxI-F and purified ToxI-S are shown. (**C**) Northern blot for individual processed forms of ToxI in *E. coli*, using a ^32^P-labelled probe.

ToxIN of *Pectobacterium atrosepticum* was the first described Type III TA system, and has a powerful Abi activity in its native host as well as *Serratia* and *Escherichia coli*, reducing replication of sensitive phage by 2 to 10 orders of magnitude^7,15^. ToxIN occurs genetically as a bicistronic operon of 5.5 36-nt repeats (ToxI) followed by the ToxN gene, with a transcriptional terminator between these two elements regulating their stoichiometry of production^7^ (Fig. 1A). ToxN is a sequence-specific endoribonuclease, and its RNase activity is required for toxicity and phage resistance^8,9^. ToxI is both a substrate and inhibitor of ToxN; the repetitive transcript is cleaved by ToxN into individual 36-nt units, which adopt a pseudoknot fold, and ToxN self-assembles with its products into a self-closing triangular 3 ToxN:3 ToxI complex^8,9^ (Fig. 1A). It is not known whether ToxI folding occurs prior to interaction with ToxN, or if the triangular complex is the main form adopted *in vivo*. ToxN inhibition is linked to assembly of the complex, and both processes can be reconstituted *in vitro*, indicating that toxin inhibition in Type III systems is robust and largely host-independent. ToxIN loci also promote their own inheritance on plasmids through the killing of plasmid-free segregants^9,16^. These self-contained and “addictive” properties likely underpin the observed horizontal dissemination of Type III TA loci between diverse bacteria^17^.

The Abi activity of ToxIN is phage-dependent; for some phage, replication is abrogated completely, while other phages are unaffected. Two other Type III TA systems also have antiphage activity; AbiQ-AntiQ of *Lactococcus lactis*^18,19^, and TenpIN of *Photorhabdus luminescens*^17,20^. Interestingly, while all three of these systems have strong, phage-dependent Abi activity, their respective resistance profiles are different. For example, the *E. coli* phage T4 is aborted by AbiQ-AntiQ but not by ToxIN^20,21^. Phages ΦS61 and ΦTE are aborted by ToxIN but not by TenpIN^20^, and ToxIN and TenpIN abort different subsets of environmental coliphage. The mechanism by which Type III TA-Abi systems are activated following phage infection to trigger a precocious lethality in an infected cell (thereby protecting the bacterial community) is not yet known. The toxin could be released passively if antitoxin levels drop sufficiently following phage infection; alternatively, a specific phage product could trigger the release of the toxin from the complex. Several studies have explored this question by sequencing phage mutants that have evolved spontaneously to bypass Type III TA-mediated Abi^20–23^. To date, escape mutations have been mapped in eight different phage, targeting different Type III TA systems and in diverse bacterial species (*P. atrosepticum*, *S. marcescens* and *Lactococcus lactis*). In most of the phage:TA pairs (ToxIN:ΦM1, TenpIN: ΦM1, AbiQ-AntiQ: c2, p008, bIL170 and coliphage 2), escape occurred through mutation of a single gene of unknown function. The observed escape genes were different in each phage, even in the case of bIL170 which encodes a homologue of the AbiQ-AntiQ escape gene of phage c2. Phage can also escape Type III-mediated Abi through molecular mimicry of the antitoxin, as seen in ΦTE ToxIN escape mutants that had acquired ToxI-like sequences which were expressed during infection^22^. Finally, a study of T4-family *Serratia* phages showed that escape of ToxIN Abi could be achieved through 1) deletion of a specific genomic region of 6.5–10 kb, 2) mutation of a gene of unknown function, or 3) mutation of the middle gene transcription co-activator AsiA.

Collectively, these findings suggest that multiple factors contribute to the activation (or escape) of Abi by Type III TA systems, that different routes of escape are possible even for a single phage, and that preferred escape mechanisms are are likely to be phage-specific.

These studies do not show whether the initial triggering of Type III Abi is active (a phage product triggers release of the toxin) or passive (a general phage-induced reduction in host transcription reduces antitoxin levels, thereby releasing the toxin). A passive activation model is supported by the observation that ΦM1, but not its escape mutant, caused ToxI levels to drop during infection^20^. However, this model fails to explain the different specificity of anti-phage activity observed among the Type III TA-Abi systems. While phage mutant sequencing studies have revealed a diversity of Abi-sensitivity genes and escape mechanisms, further understanding is limited by the paucity of knowledge on the escape genes themselves. An important complementary approach to these mutant studies is to explore the properties of the ToxIN complex itself, because features of the synthesis, assembly or degradation of the complex are potential nodes for sensing or interacting with incoming phages. Consequently, these processes are likely to influence the capacity of the system to mount an effective Abi response.

In this study, we have investigated properties of the *P. atrosepticum* ToxIN complex *in vitro* and *in vivo*, with a view to understanding the events following infection by phage that activate abortive infection. We examine the half-life and turnover of ToxI, and provide evidence that the ToxI antitoxin is partially structured even in the absence of ToxN. Finally, we show a link between complex stability and effectiveness of the Abi response in two different bacterial species.

## Results

### The ToxI RNA has a half-life of 3.2–4 minutes

ToxIN can stabilise plasmids through post-segregational killing (PSK), indicating that ToxI is probably less stable than ToxN. While PSK of plasmid-free segregants has no time limit, for Abi the toxin must be released before the phage can replicate productively. We therefore wished to define the half-life of ToxI RNA *in vivo* to determine if passive activation is a possibility. The stability of the full-length ToxI transcript (ToxI-F) was assessed by Northern blot, using total RNA samples extracted from transcription-arrested exponential-phase *E*. *coli* DH5α cells carrying a high-copy vector with the full *toxIN* locus. As shown (Fig. 1B), the full-length ToxI RNA (ToxI-F) was present together with smaller products, and was degraded over the course of the experiment with a half-life of 3.2 min. It was not possible to see individual degradation products, or single ToxI repeats (ToxI-S), due to the RNA purification method used (which excludes RNAs of <100 nt) and the resolution of the agarose gel. To visualise these products, a further Northern blot was performed using a ^32^P-labelled probe and hot phenol-purified RNA (Fig. 1C), run on a denaturing acrylamide gel. At t = 0 the majority of the ToxI RNA was present as the full-length transcript and intermediate-sized processed forms. ToxI-S repeats were visible from 7.5 min after rifampicin addition. The half-life of ToxI-F was 4 min, and the half-life of ToxI-S (calculated from the peak at t = 20) was 13 min, although this measurement may not represent the half-life in normal cells where transcription is not arrested. A surprising observation in this experiment was that intermediate processed lengths of ToxI were very abundant at t = 0 even while ToxI-S was not detected; this raises the possibility that under high expression conditions ToxN constantly cleaves fresh ToxI-F rather than staying in complex with its product, ToxI-S. The effect of ToxI-S on cleavage of ToxI-F by ToxN was examined next.

### ToxIN is a dynamic complex

**Figure 2:**
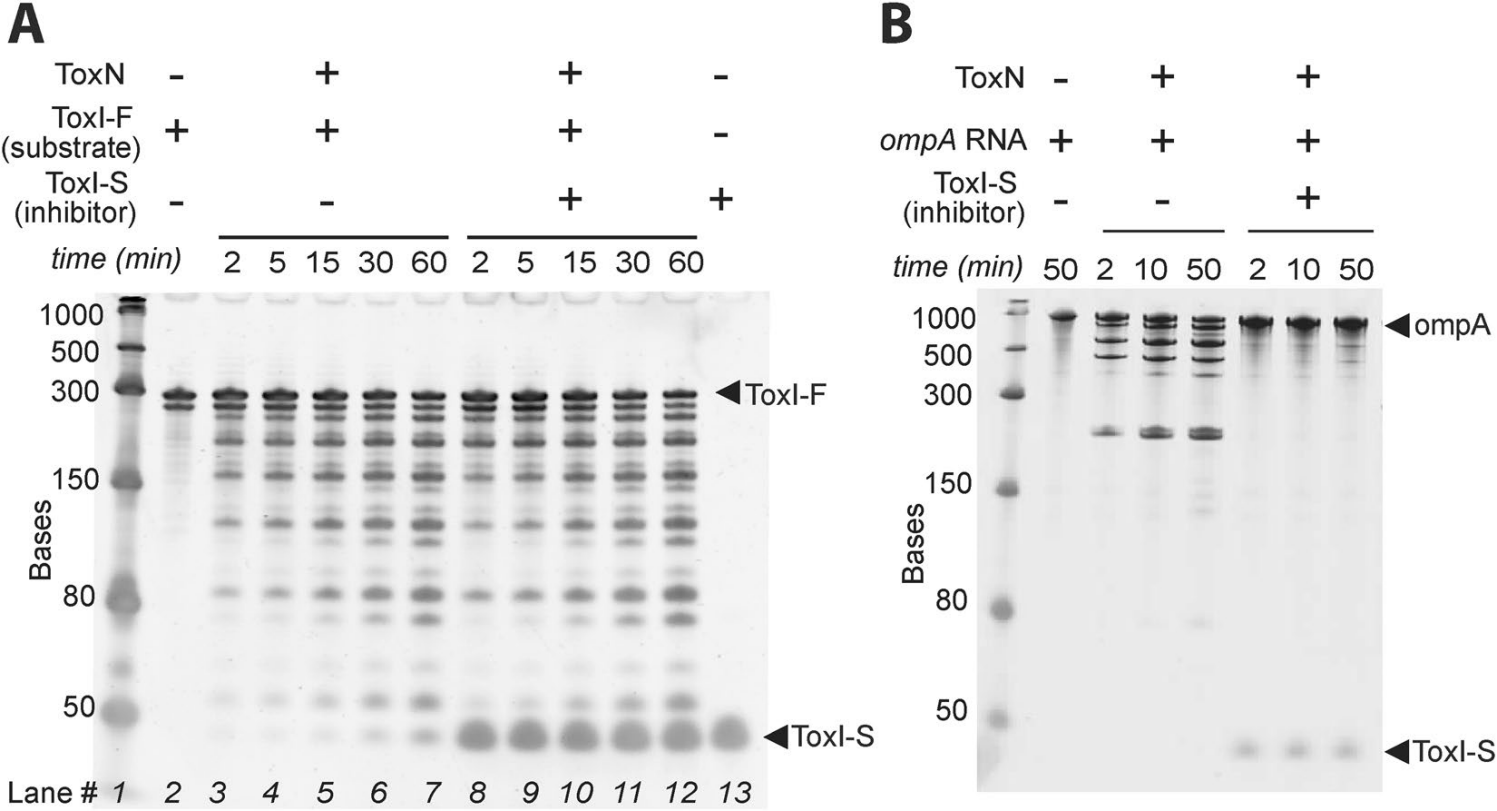
(**A**) Effect of ToxI-S on ToxN cleavage of ToxI-F. Purified ToxN was either used directly, or was incubated with ToxI-S in a 1:4 molar ratio for 15 minutes, prior to addition of the ToxI-F substrate. Samples taken at the indicated time points were quenched in RNA loading buffer and immediately flash-frozen in liquid N_2_. ToxI-F was cleaved into a ladder of bands and the rate appeared the same in both reactions. (**B**) Control reactions with an *ompA* RNA substrate, using the same ToxN and ToxI-S preparations as in A, showed the expected inhibition.

We showed previously that ToxN cleaves RNAs *in vitro*, and can be inhibited by ToxI-F and ToxI-S^9^. To investigate the effect of ToxI-S on ToxN activity against ToxI-F, *in vitro* stop-point RNA degradation assays were performed (Fig. 2A). ToxN and ToxI-S were incubated together for fifteen minutes prior to addition of ToxI-F to allow the ToxIN complex to self-assemble as shown *in vitro*^9^. ToxI-F was cleaved by ToxN into a ladder of bands over the course of the reaction, although some full-length transcript still remained at the end (Fig. 2A, lanes 3–7). Note that ToxI-F contained RNA of two different lengths (Fig. 2A, lane 2), because the *toxIN* locus hairpin terminator was present in the *in vitro* transcription template and caused premature termination in some, but not all, transcripts (see Fig. 1A). ToxI-S was added at a fourfold molar excess over ToxN and, unexpectedly, did not appear to affect the rate of ToxI-F transcript degradation (Fig. 2A, lanes 8–12). This effect was not due to the ToxN or ToxI-S samples being defective, as a parallel *in vitro* inhibition assay with *ompA* RNA as the substrate showed the expected ToxN inhibition (Fig. 2B). This experiment shows that cleavage of ToxI-F by ToxN proceeds even in the presence of a large excess of ToxI-S. A possible explanation for this observation is that ToxI-F can displace ToxI-S from ToxN, while other RNAs cannot.

### ToxI spontaneously folds into a pseudoknot independently of ToxN

**Figure 3:**
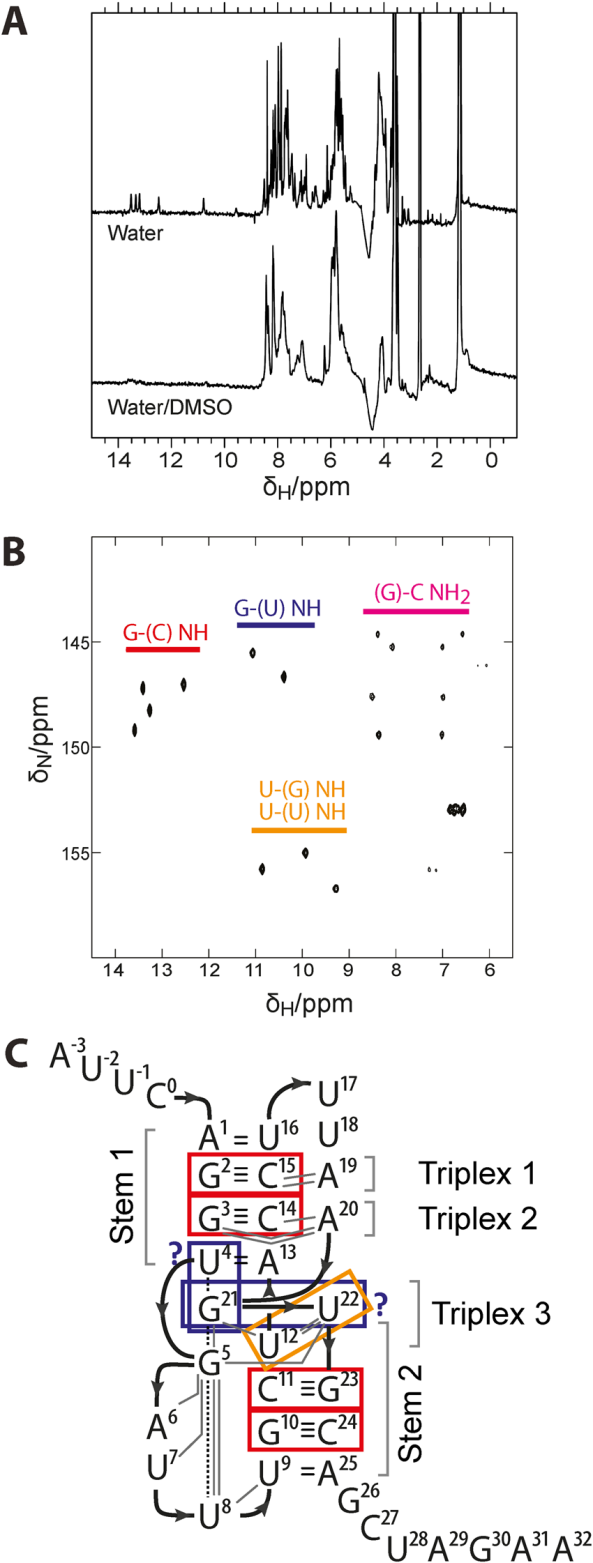
ToxI is partially structured in solution. (**A**) 1D ^1^H spectra collected with ToxI in either native or denaturing (50% DMSO) buffer. The chemical shifts corresponding to base-paired imino protons are in the region of 10–14ppm. (**B**) 2D [^1^H, ^15^N]-HSQC spectrum of ^15^N-ToxI with predicted base assignments. (**C**) Schematic of internal bonding within the ToxI crystal structure, with key structural features, and interactions observed by NMR, indicated. The ToxI pseudoknot schematic shows the path of the RNA backbone in bold arrows, hydrophobic stacking interactions as dashed lines, Watson-Crick base pairs as two (A:U) or three (G:C) horizontal bars, and additional hydrogen bonds as grey lines, and is adapted from Blower *et al*.^8^.

While the ToxIN complex can self-assemble and is stable *in vitro*^9^, ToxI processing is surprisingly dynamic. ToxN appears to target and rapidly turn over fresh ToxI-F both *in vivo* and *in vitro*. Of relevance to both complex assembly and turnover is the question of whether ToxI can fold independently. ToxN may first encounter ToxI as “beads on a string” - a series of complete pseudoknots interspersed by ToxN cleavage sites. Alternatively, ToxI may fold only after contact with its partner enzyme, ToxN. NMR experiments with native and ^15^N-labelled ToxI-S were performed to explore this question. For both sets of experiments, ToxI-S was purified by phenol-chloroform extraction of ToxIN complex (itself purified following overexpression in *E. coli*), then denatured by heating and allowed to cool slowly. It was not possible to purify sufficient amounts of ToxI-S for NMR analysis without denaturation of the complex. The ^1^H spectrum of unlabelled ToxI-S showed five signals in the 10.5–14 ppm region (Fig. 3A), which are characteristic of imino protons found in base pairs^24^. These signals were absent for ToxI-S in denaturing buffer, confirming that they are indeed due to the internal structure of ToxI-S.

The ^15^N-ToxI-S samples showed nine imino proton signals and eight amino proton signals in a [^1^H,^15^N]-HSQC experiment (Fig. 3B). Probable base pair types responsible for each resonance were assigned based on reported ^15^N chemical shift values for G and U^25^. As shown, four signals from G imino groups involved in G:C base pairs were observed, together with two signals from G imino groups in G:U base pairs, and three from U imino groups in either G:U or U:U pairs. The four G:C signals, and one of the U:U/U:G signals, were also observed in the 1D spectrum (Fig. 3A). The four G:C signals are likely to represent the four canonical Watson-Crick base pairs of the ToxI pseudoknot, which are derived from both base-paired stems (Fig. 3C). The five other imino signals detected are from non-canonical G:U or U:U base pairs. Typically, non-canonical base pairs are identified by NMR using double-labelled samples, so their expected chemical shifts in [^1^H,^15^N]-HSQC spectra are poorly defined. Several different types of G:U and U:U base pairs are possible, and these pairs can involve one or two imino groups. No A:U base pairs were observed in the HSQC spectrum, however it may be that A:U pairs can form, but are too unstable for detection by NMR.

Overall, the NMR data are consistent with ToxI folding spontaneously into a structure with four G:C base pairs, two G:U base pairs and one or two U:U base pairs. A model is shown for how these pairs may fit with the ToxI-S structure observed in the crystallographic complex (Fig. 3C): both base-paired stems of the pseudoknot are partially formed, and the non-canonical G:U and U:U base pairs are formed in the central region. Note that Triplex 3 of ToxI-S does not shield any G imino protons, so the G (U) resonances seen in this experiment do not indicate the spontaneous formation of Triplex 3. These results show that some features of the mature ToxI pseudoknot, in particular the Watson-Crick G:C base pairs from Stem I and Stem II, can form spontaneously in solution.

### Reduced ToxIN complex stability enhances phage resistance range in *P. atrosepticum*

We previously reported a ToxN mutant, ToxN-Y115A, that showed reduced response to ToxI^8^. The toxicity of this mutant was not rescued by co-overexpression of ToxI from a separate plasmid, however this variant could be cloned as part of the full *toxIN* locus on a medium-copy plasmid, and showed wild-type activity against two phages^8^. To examine the effect of this destabilising mutation on ToxIN activity against phage, the mutant was tested against a suite of 14 uncharacterised *P. atrosepticum* phages isolated from treated sewage effluent.

**Figure 4:**
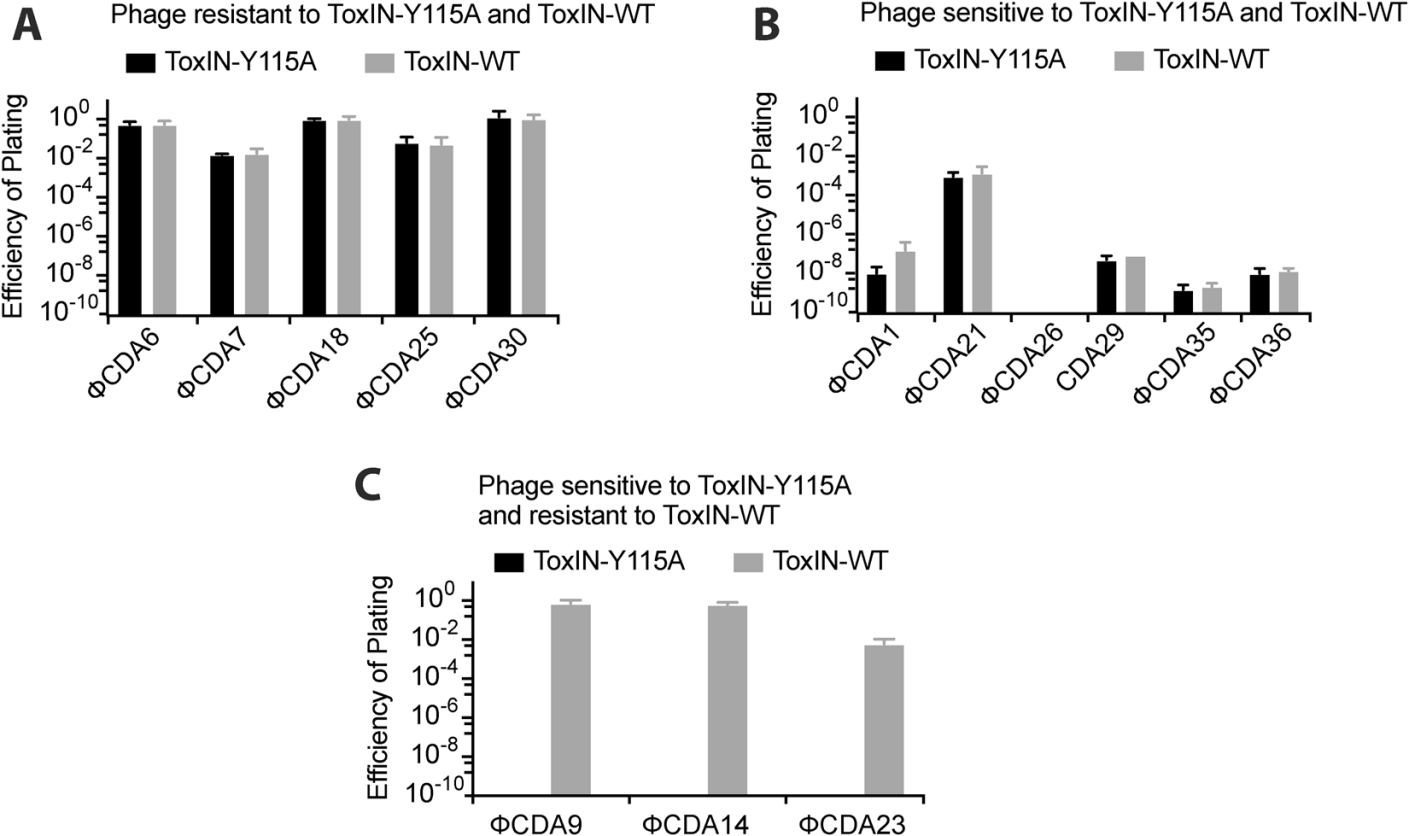
Effect of less stable ToxIN-Y115A mutant on abortive infection of *P. atrosepticum* phage isolated from sewage effluent (**A**) Phage resistant to both ToxIN-Y115A and ToxIN-WT. (**B**) Phage aborted by both ToxIN-Y115A and ToxIN-WT. (**C**) Phage aborted by ToxIN-Y115A and resistant to ToxIN-WT.

The bacteriophage tested against ToxIN and ToxIN-Y115A fell into three categories; those insensitive to both systems (5 phage, Fig. 4A), phages that were aborted by both systems (6 phage, Fig. 4B), and phages that were aborted by ToxIN-Y115A but not by ToxIN-WT (3 phage, Fig. 4C). We did not identify any phage that were aborted more efficiently by ToxIN-WT than by the unstable mutant. This indicates that the stability of the ToxI-ToxN interaction is an important aspect of the Abi activity of ToxIN, and, in some cases, reduced stability enables the system to abort otherwise insensitive phages.

### Unstable ToxIN can abort evolved mutant ToxIN-insensitive phages in Serratia

We then wished to determine whether an “escape” phage that had evolved heritable resistance to ToxIN could still bypass the (conditionally) stronger Abi by ToxIN-Y115A. For these experiments we tested eight previously isolated escape mutants of the *Serratia* phage ΦCHI14^23^. Five of these phage have been sequenced completely, and escape ToxIN by mutation of *asiA* (ΦCHI14b, ΦCHI14f) or *orf84* (ΦCHI14e), or through a large genomic deletion (ΦCHI14a, ΦCHI14c); the other four have not been fully sequenced but were shown by PCR to contain *asiA* mutations^23^. Interestingly, all of the ΦCHI14 ToxIN escape phages tested, collectively representing at least three distinct escape mechanisms, were still aborted by ToxIN-Y115A (Table 1). Two of these escape phages – a deletion mutant and an *orf84* mutant – gave rise to “double escape” mutants able to resist both systems when passaged on ToxIN-Y115A (Table 1).

**Table 1:** Activity of ToxIN-Y115A against ToxIN escape phage.

*Phage* ^*a*^	*Mutation*	*Efficiency of plating*	
		ToxIN-Y115A-FLAG	ToxIN-FLAG
ΦCHI14	None	<9.5 × 10^−9^	1.6 × 10^−8^
***Escape phage***			
**ΦCHI14a**	7.6 kb deletion	3.5 × 10^−7^	0.81
**ΦCHI14b**	*asiA* E71 → Stop	<2.2 × 10^−6^	0.15
**ΦCHI14c**	10.1 kb deletion	<2.1 × 10^−6^	1.06
**ΦCHI14e**	*orf84* E66D	8.0 × 10^−6^	0.48
ΦCHI14f	*asiA* frameshift	8.2 × 10^−7^	0.48
ΦCHI14q	*asiA* frameshift	<8.5 × 10^−8^	0.34
ΦCHI14s	*asiA* frameshift	<2.2 × 10^−8^	0.08
ΦCHI14w	*asiA* V13G	<8.0 × 10^−8^	0.38
***Double-escape phage***			
ΦCHI14a-m1	7.6 kb deletion + unknown	0.02	0.14
ΦCHI14e-m1	*orf84* E66D + unknown	0.12	0.26

^**a**^Phage where mutations were identified by whole-genome sequencing are indicated in bold. Other mutations were determined by PCR.

## Discussion

All Abi systems require a suppression mechanism to prevent the toxin from harming the cell during normal growth. The use of a pseudoknotted RNA antitoxin (rather than another protein) in ToxIN, AbiQ-AntiQ and other systems represents a structurally-elegant solution to this problem. Although the inhibitory activity of ToxI has been well-defined, the mechanism behind the other half of its role – to release ToxN on phage infection – is poorly understood. We have explored the turnover and folding of ToxI with a view to understanding how the complex is triggered following phage infection.

The unprocessed ToxI, ToxI-F, was shown to have a half-life of 3.2–4 min; within the normal range for an mRNA in *E. coli*^26^. The half-life of ToxI-S was longer at 13 min. However, because its levels peaked 20 minutes after transcription arrest, this cannot be taken as a reliable measure of its half-life in normal cells. Typical phage latent periods range from 20–60 minutes^27^, with host transcription shut down early in the replication cycle. Though the observed half-life of ToxI-F is consistent with passive release of ToxN, further experiments will be required to determine if this process is a general activation mechanism for Type III-mediated Abi. Furthermore, the choreography of the process would also depend on the timescale of the lytic cycle of the infecting phage, and on the amount of antitoxin present initially. A passive activation model would predict that increasing ToxI production above a certain threshold would decrease the Abi activity of the system; interestingly, a study of AntiQ-AbiQ showed that Abi of phage P008 is abrogated by increasing the number of antitoxic repeats in the locus from 2.8 to 3.8^28^. The same study also showed a reduction in Abi efficiency when one *antiQ* repeat was deleted. The effect of increased antitoxin repeat numbers on ToxIN Abi has not been tested, while constructs with two or three repeats deleted displayed slightly higher Abi activity^15^. More work is needed to explore Type III-mediated Abi of phages with different latent periods and antitoxin levels, in order to either confirm or refute the passive activation model.

Analysis of ToxI *in vivo* revealed an unexpected pattern where the RNA was present primarily as ToxI-F and intermediate-sized forms, suggesting that ToxN is diverted to fresh unprocessed ToxI-F when available – a result that we also demonstrated *in vitro*. It is formally possible that ToxN has a higher affinity for uncleaved ToxI-F (which could bind across both RNA interaction surfaces of the protein) than for ToxI-S, which would allow ToxI-F to displace ToxI-S from the complex and drive continuous turnover of the antitoxin. However, we were unable to measure this directly (data not shown). The ToxIN complex is stable in solution and its formation is linked to inhibition, but it appears that this complex is not the dominant form of ToxIN *in vivo*, at least under high expression conditions. Instead, ToxN is preferentially diverted to fresh substrate, which is rapidly turned over. The dynamic nature of this system *in vivo* may render it more physiologically plastic, thereby enabling enhanced responsiveness to environmental insults, such as phage infection.

The ToxI antitoxin can fold into a pseudoknot spontaneously, as G:C base pairs from both stems of the structure were observed by NMR. It was not possible to compare the solution structure of spontaneously folded ToxI-S with that of the mature ToxN-associated form because we were unable to purify sufficient native ToxI-S for analysis, and ToxI in complex with ToxN generated only weak NMR signals. ToxI has two G:C base pairs in each of its pseudoknot stems (See Fig. 3C), but relatively few of these nucleotides overall (6 C and 8 G, compared to 10 A and 12 U nucleotides). These G:C base pairs, which have little potential for mispairing, may function to “lock in” a productive folding pathway early on, and subtle ToxN-induced structural changes could then produce the final mature ToxI. Other antitoxins of the ToxI family also utilise G:C base pairs in their pseudoknot stems but have relatively few G and C nucleotides overall^17^, and this property is also seen in the structure of the only characterised CptI antitoxin^29^. We suggest that robust folding of the pseudoknot topology may have been selected for over the evolutionary history of ToxI.

Abi activity was increased in a ToxIN mutant with reduced ToxI-ToxN binding. The effect was phage dependent – 3 of the 9 *P. atrosepticum* phages that were not affected by WT ToxIN were aborted very strongly by the unstable mutant, with escape EOP values of ~10^−9^. Of the ΦChi14 “escape” phages that had evolved to bypass ToxIN in *Serratia*^23^, all eight were still aborted by ToxIN-Y115A, and two gave rise to escape mutants able to bypass this system as well, though the nature of the secondary escape mutation is unknown. The fact that an Abi-sensitive phage can sequentially evolve resistance to ToxIN, and then to a more effective variant of this system, illustrates the powerful antagonistic co-evolution that is constantly played out between bacteria and their viral predators. Under lab conditions of growth, ToxIN-mediated Abi was an “all-or-nothing” phenotype with these particular phages; the ToxIN-Y115A mutation appeared to increase the likelihood of an insensitive phage being aborted very strongly, but did not give rise to intermediate phenotypes or increased resistance to sensitive phage. An Abi-promoting point mutation has also been described for the *antiQ-abiQ* system; this mutation increases Abi activity against P008 by 2 orders of magnitude^28^. In this case the mutation, AntiQ-G32A, is predicted to disrupt the pseudoknot structure of the antitoxin and reduce antitoxicity, though this was not tested directly. The stability dependence of Abi therefore seems to be a common feature of bifunctional Type III TA-Abi systems.

ToxIN is a very powerful Abi system with multiple features that contribute to its activity. We have shown here that the complex constantly turns over when excess ToxI is present, that the ToxI antitoxin can fold spontaneously, and that reducing the stability of the toxin-antitoxin interaction can alone determine a switch from no Abi to an Abi phenotype of over 9 orders of magnitude. This new information complements recent studies that have identified specific phage genes of unknown function as candidate ToxIN or AbiQ-AntiQ sensitivity factors.

**Figure 5:**
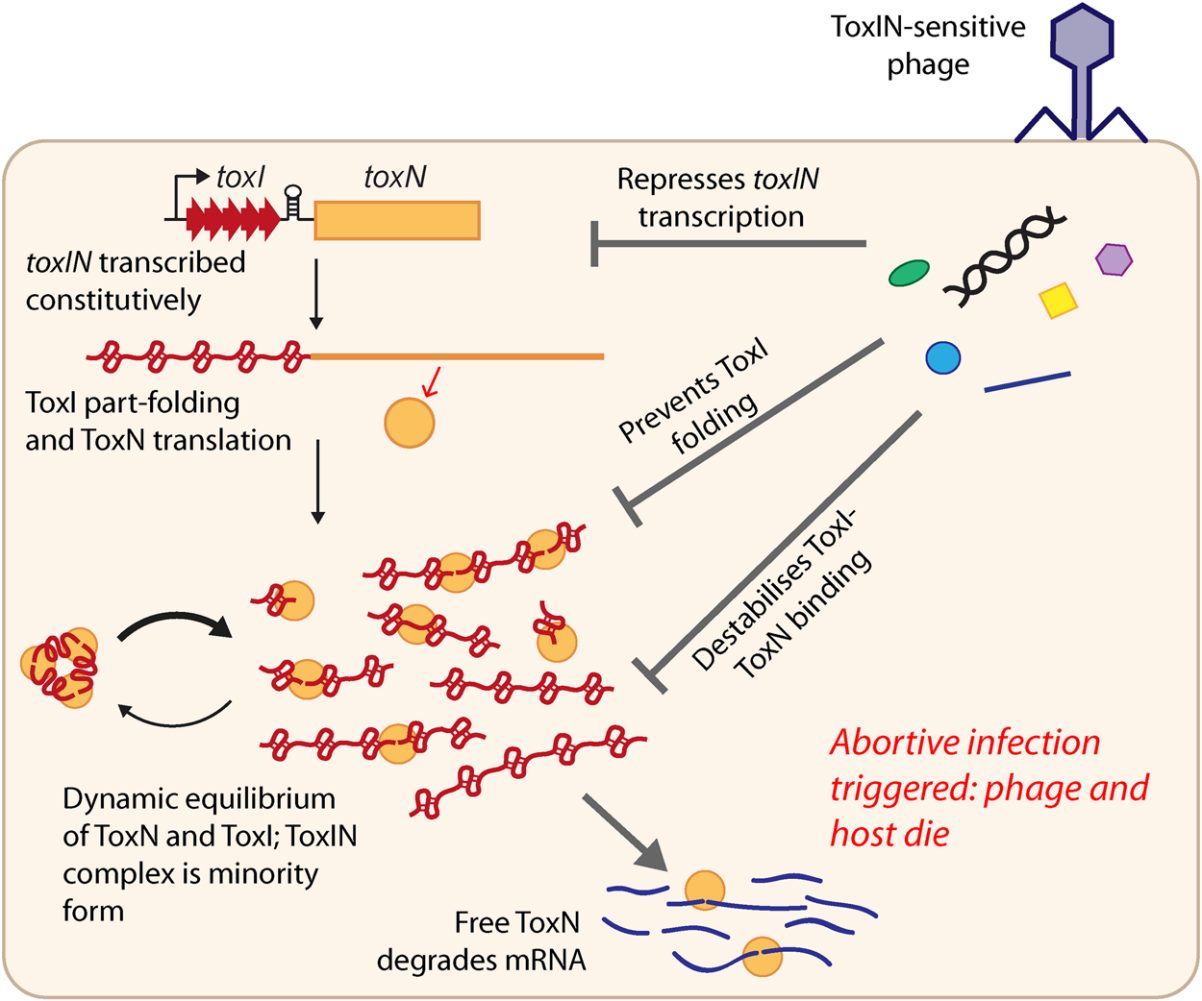
Model for activity of ToxIN against incoming phage. Both components are transcribed constitutively, and individual repeats of ToxI spontaneously fold into RNA pseudoknots. ToxN cleaves ToxI-F and can induce structural changes in ToxI to assemble with its products into a stable, trimeric complex. Fresh ToxI-F diverts ToxN from the complex so there is a dynamic equilibrium between ToxIN complex, and ToxN bound to fresh or partially cleaved ToxI-F. Under high expression conditions this equilibrium is shifted to the right as a large excess of ToxI-F is present. Phage infection can result in ToxN release through the repression of *toxIN* transcription, through preventing the folding of ToxI and therefore its antitoxin capacity, or through destabilising ToxI-ToxN binding. As the trimeric ToxIN complex is not the predominant form under conditions of high expression, activation is not likely to be through direct disassembly of pre-formed ToxIN complexes. Free ToxN cleaves cellular RNAs, and if a sufficient amount of ToxN is released then the bacterial cell, and its phage parasite, will die.

A tentative model for ToxIN Abi activity based on these new findings, along with previous results, is presented in Fig. 5. ToxI and ToxN are expressed continuously, ToxI in excess, and individual pseudoknots of ToxI form along the full-length transcript. ToxI can self-assemble an inactive ToxIN complex, but fresh ToxI-F can displace mature ToxI-S, meaning that ToxN is continuously binding, cleaving and dissociating from fresh ToxI under high expression conditions. Bacteriophage infection causes ToxN release through either 1) suppression of *toxIN* transcription, 2) prevention of ToxI folding, or 3) prevention of interaction of ToxI and ToxN (for example, by binding ToxN, or by promoting degradation of ToxI). Because the ToxIN complex appears to be a minority form *in vivo*, it is unlikely that a phage product would trigger Abi solely through disassembling pre-formed ToxIN complexes. Sufficient release of ToxN prior to completion of the phage lytic cycle will result in dramatic damage to the bacterial cell’s mRNA pool, and the death of the host cell and the phage invader. We suggest that ToxN release may be triggered by different stimuli in different phage, with passive release due to transcription arrest the ‘default’ outcome for slowly-replicating phage not encoding ToxN inhibitors, and other factors determining the outcome in phage with short lytic cycle times. Type III-mediated Abi appears to be an extraordinarily subtle process, with multiple inputs from both the host and the phage influencing a binary outcome of strong resistance vs complete sensitivity. The dynamic nature of the ToxIN system provides multiple routes for disruption by viral products. Our results highlight how a self-contained module of toxic protein and antitoxic RNA can act as a highly sensitive and effective “suicide” based phage resistance system in populations of bacteria.

## Materials and Methods

### Bacterial strains, bacteriophage, and growth conditions

Bacterial hosts and phage used in this study are listed in Table 2. Bacterial strains were grown on liquid growth media Luria-Broth (LB). Solid LB-agar (LBA) plates and top lawns were made with a 1.5% and 0.35% w/v agar (Melford Laboratories), respectively. *Escherichia coli* strains were grown at 37 °C. *Serratia* strains were grown in liquid and solid media at 30 °C. *P. atrosepticum* strains were grown on liquid at 30 °C and solid media at 25 °C. Bacterial growth was monitored by measuring optical density at 600 nm using a Unicam Heλios spectrophotometer to give OD_600_. If required, media were supplemented with antibiotics at the appropriate concentration.

**Table 2:** Bacterial strains, bacteriophages and plasmids used in this study.

*Name*	*Description*	*Source*
**Bacterial strains**		
*Escherichia coli* DH5α	K-12 strain: F^−^ Φ80*lacZ*ΔM15 Δ*(lacZYA-argF)* U169 *recA*1 *endA*1 *hsdR*17(r_K_^−^ m_K_^+^) *phoA supE*44 λ^−^ *thi*-1 *relA*1 *gyrA*96	Invitrogen
*Pectobacterium atrosepticum* SCRI1043	Wild-type environmental isolate	^33^
*Serratia marcescens* ATCC39006	Wild-type environmental isolate, carbapenem+, prodigiosin+	^34^
**Bacteriophage**		
ΦCDA [1–29]	Uncharacterised environmental *P. atrosepticum* phage, sewage isolate	This study
ΦCHI14[a-x]	Escape mutants of *S. marcescens* 39006 phage ΦCHI14	^23^
**Plasmids**		
pFLS6	*toxIN*-Y115A-FLAG in pBR322, Ap^R^	^8^
pFLS90	*toxIN* locus in pBS KSII+, Ap^R^	This study
pMJ4	*toxIN*-FLAG in pBR322, Ap^R^	^15^
pTA46	*toxIN* in pBR322, Ap^R^	^7^
pTA47	*toxIN*-FS in pBR322, Ap^R^	^7^
pTA110	*toxI* in pBS KSII+ for antisense transcription, Ap^R^	^9^
pTRB57	*toxIN*-FS-FLAG in pBR322, Ap^R^	^15^

### ToxI half-life measurement

*E. coli* DH5α carrying the *toxIN* plasmid pFLS90 (Table 2) was grown in LB at 37 °C to an OD600 of 0.8. Rifampicin was then added at a final concentration of 250 μg/ml to stop transcription. Samples of 2–3 OD equivalents (approx 2–3 * 10^9^ cells) were immediately added to two volumes of ice-cold 5% phenol, 95% ethanol, and stored at −80 °C prior to RNA extraction. For the DIG probe Northern blot, RNA was first extracted using an RNeasy Mini Kit (Qiagen) according to the supplied instructions, and 8 μg total RNA from each time-point was denatured at 80 °C for 10 minutes in NEB RNA loading dye and loaded onto a 1× MOPS, 1.5% agarose gel. Standards of 20 ng each of ToxI-F and ToxI-S were used as controls. A DIG-labelled antisense ToxI-F probe was generated by *in vitro* transcription using DIG RNA labelling mix (Roche) with a PCR product template generated from pTA110 using primers M13-20 (5′-GTTTTCCCAGTCACGAC-3′) and TRB57 (5′-TTTGAGCTCAAGGTGATTTGCTACCTTTAAG-3′). The Northern blot was then performed according to the DIG user manual (Roche). For the Northern blot using ^32^P, total RNA was purified as described^30^ and treated with DnaseI (Qiagen), then re-extracted with chloroform and ethanol precipitated. 6 μg total RNA from each time-point sample was denatured in NEB RNA loading buffer and electrophoresed on a 6% acrylamide TBE-Urea gel (National Diagnostics). The probe was produced by labelling a single-stranded antisense ToxI DNA oligonucleotide (FS198, Sigma) with 0.74 Mbq ^32^P-ATP using T4 polynucleotide kinase (NEB) at 37 °C 45 minutes, and purified using a SigmaSpin sequencing reaction clean-up column. RNA samples were transferred to a positively charged nylon membrane by electroblotting at 4 °C for 90 minutes 50 V in 1× TBE, and the RNA was cross-linked for 4 minutes each side with a 3 UV cross-linker. Pre-hybridisation and hybridisation steps were carried out in Church’s Buffer (0.5 M NaPO4 pH 7.2, 7% SDS, 1 mM EDTA) for 1 hr at 42 °C, with 50 pmol of the labelled probe added at the hybridisation step. The membrane was then washed for 15 minutes each with 5 × SSC 0.1% SDS, then 1 × SSC 0.1% SDS, followed by 0.5 × SSC 0.1% SDS. X-ray film was exposed to the blot for 24 hours at −80 °C. Northern blot images were analysed using ImageJ to measure band intensities, and half-life values were calculated by fitting the measured intensities to an exponential function.

### *In vitro* RNase assays

ToxN, ToxI-F and ToxI-S samples were prepared as described^9^. 10 pmol ToxN, with or without 40 pmol ToxI-S, was made up to a volume of 12.75 μl in RNA buffer 2 and incubated 15 minutes at 25 °C to allow binding of ToxN and ToxI-S. Reactions were started by addition of 2.25 μl (~14.5 pmol) ToxI-F transcript and incubated at 25 °C for 60 minutes, with time-point samples of 3 μl each taken over the course of the reaction.

### ToxI purification and NMR

The native ToxIN complex was overexpressed and purified as described^8^. ^15^N-labelled ToxIN_Pa_ was generated in the same way, except that the expression medium was M9 minimal medium containing ^15^NH_4_Cl as the sole nitrogen source. Following anion exchange chromatography, fractions that were observed to contain either ToxIN complex or ToxI were pooled and the RNA in the sample was purified by phenol-chloroform extraction as described^30^ and precipitated with ethanol overnight. The RNA pellet was washed with 70% ethanol, resuspended in 50 ml 50 mM sodium phosphate pH 7.2, and dialysed against 1 L of 50 mM sodium phosphate pH 7.2 overnight to remove excess salt. The dialysed sample was recovered, and was concentrated in a vivaspin 5 kDa MWCO concentrator. Purified native and ^15^N-labelled ToxI samples were denatured at 90 °C for two minutes in a heat block and slowly cooled to ambient temperature by switching off the heat block. The samples were prepared for NMR analysis by adding 20 μM 3,3,3-trimethilsilylpropionate and 10% D_2_O (Sigma) to a final volume of 550 μl. NMR spectra were taken in 5 mm Ultra-Imperial grade NMR tubes (Wilmad), with final ToxI concentrations of 191 μM (unlabelled ToxI) and 240 μM (^15^N-labelled ToxI). Spectra were recorded at 288 K on a Bruker DRX500 spectrometer equipped with a z-shielded gradient triple resonance probe. 1D ^1^H and 2D [^1^H, ^15^N]-HSQC spectra were recorded using standard procedures^31^.

### Isolation of novel bacteriophage

*P. atrosepticum* phage, labeled ΦCDA, were isolated by enrichment from treated sewage effluent. To prepare a phage enrichment, 25 ml of sewage water were filter sterilized using a 0.20 μm microfilter and added to 25 ml of 2X LB media. Afterwards, 500 μl of an overnight culture of bacteria was added to flasks and incubated at 225 rpm and 30 °C overnight. The following day, a 1 ml sample of the culture was mixed with 100 μl of chloroform and vortexed vigorously. The vortexed sample was spun down at 16,000 × g for 2 min and a dilution series of the supernatant was plated on the appropriate host. Any resulting plaques were isolated and immersed in 200 μl of phage buffer and 40 μl of chloroform. The contents were vortexed briefly and spun down at 16,000 × g for 2 min. The supernatant was then titrated by adding 10 μl from a dilution series to 250 μl of bacterial culture and 3 ml of top LBA. The plates were incubated overnight at the appropriate temperature. Plates with near confluent lysis were used to generate phage lysates.

### Efficiency of Plating measurements

Efficiency of plating (EOP)^32^ was calculated using the equation: titer on test strain/titer on control strain. EOP measurements were used to determine the susceptibility of phage to ToxIN. *P. atrosepticum* test and control strains carried the plasmids pFLS6 (ToxIN-Y115A-FLAG), pMJ4 (ToxIN-FLAG) or pTRB57 (ToxIN-frameshift-FLAG). For *S* 39006 the test strains contained the plasmid pTA46 (ToxIN-WT) or pFLS6 (ToxIN-Y115A-FLAG). The control *S* 39006 strain contained plasmid pTA47 (ToxIN-FS).

### Isolation of ΦCHI14 phage double escape mutants

ΦCHI14 escape phage used are described in Table 2. ToxIN-Y115A escape phage lysates were isolated as described^23^ from plates containing a lawn of *S* 39006 pFLS6.

### Data availability statement

All data generated or analysed in this study are included in this manuscript.
